# Chronic social defeat induces long-term behavioral depression of aggressive motivation in an invertebrate model system

**DOI:** 10.1371/journal.pone.0184121

**Published:** 2017-09-14

**Authors:** Jacqueline Rose, Jan Rillich, Paul A. Stevenson

**Affiliations:** Institute for Biology, Leipzig University, Talstrasse, Leipzig, Germany; University of Cologne, GERMANY

## Abstract

Losing a fight against a conspecific male (social defeat) induces a period of suppressed aggressiveness and general behaviour, often with symptoms common to human psychiatric disorders. Agonistic experience is also discussed as a potential cause of consistent, behavioral differences between individuals (animal “personality”). In non-mammals, however, the impact of single agonistic encounters typically last only hours, but then again studies of repeated intermittent defeat (chronic social defeat) are seldom. We report the effect of chronic social defeat in adult male crickets (*Gryllus bimaculatus*), for which all known behavioral effects of defeat last only 3 h. Firstly, after 48 h social isolation, crickets that experienced 5 defeats at 24 h intervals against the same, weight-matched opponent exhibited suppressed aggressiveness lasting >24 h, which was still evident when the animals were matched against an unfamiliar opponent at the last trial. Secondly, this longer-term depression of aggression also occurred in 48 h isolated crickets that lost 6 fights at 1 h intervals against unfamiliar opponents at each trial. Thirdly, crickets isolated as larvae until adult maturity (>16 days) were significantly more aggressive, and less variable in their aggressiveness at their very first fight than 48 h isolates, and also significantly more resilient to the effects of chronic social defeat. We conclude that losing an aggressive encounter in crickets has a residual effect, lasting at least 24 h, that accumulates when repeated defeats are experienced, and leads to a prolonged depression of aggressive motivation in subordinates. Furthermore, our data indicate that social interactions between young adults and possibly larvae can have even longer, possibly lifelong influences on subsequent behavior. Social subjugation is thus likely to be a prime determinant of inter-individual behavioral differences in crickets. Our work also opens new avenues for investigating proximate mechanisms underlying depression-like phenomena.

## Introduction

Social experience, particularly agonistic encounters between conspecifics, has profound effects on the subsequent behavior of all animals critically tested. Whereas winning a contest (social dominance) generally tends to enhance aggressiveness and the chances of winning subsequent encounters, losing (social defeat) is typically followed by a prolonged period of depressed aggressiveness and lowered chances of winning [1, 2]. Social defeat is also considered to be a major stressor [3], that in mammals induces depression like symptoms [4], involving reductions in locomotory behaviors and changes in brain chemicals, that become increasingly severe and persistent with repeated intermittent defeats (chronic social defeat, [5]). Investigations of the consequences of social defeat in a variety of animals are thus receiving increasing attention as potential models for understanding human psychiatric disorders such as depression and post-traumatic stress [6,7,8].

Although still under debate [9], evidence is also mounting that agonistic experience is both a cause and consequence of inter-individual differences in behavior, i.e. “personality”, in vertebrate and invertebrate animals alike (review: [10]). For example, tadpoles reared in isolation and without predatory cues no longer show inter-individual behavioral differences [11], while changes in dominance status erodes putative personality traits of exploration and activity in crickets (*Teleogryllus oceanicus*, [12]). Our recent work on male crickets (*Gryllus bimaculatus*) has also revealed significant differences in locomotory and exploratory behavior between aggressive winners and submissive losers of previous contests [13]. Interestingly, these behavioral differences were also largely evident even before the contest, in individuals that were previously socially isolated for 48 h. This is surprising since all known behavioral effects of previous agonistic experience in crickets should have abated after this period of isolation: winners exhibit hyper-aggressiveness for no longer than 20 min after victory [14], while submissive losers typically regain their aggressiveness 0.5–3 hours after defeat [15–17]. However, since agonistic interactions can influence neurogenesis and gene expression in the house cricket *Acheta domesticus* [18, 19], longer term behavioral effects of social experience in crickets may have escaped the attention of previous investigators.

In this paper, we test the hypothesis that chronic social defeat may have longer term effects on the aggressive behavior of crickets than single defeats, as demonstrated in mammals [5, 7]. Three defeats were previously noted to extend the loser effect in crickets from 3 to 6 hours [16], but the phenomenon has not been studied systematically, and it is not known if more defeats have a greater effect. In dyadic contests between male fruit flies, the loser effect could be extended from several hours after a single defeat to a day after multiple defeats against the same opponent [20]. In this study, lengthening of the duration of the loser effect was thought to partly result from losers adapting their fighting strategy towards familiar opponents, as well as changes to a more effective fighting strategy in winners. In the present study, we sought to eliminate potential effects of individual recognition and changing winner strategies, for example by swapping opponents at successive loser trials. Our data reveal that chronic social defeat alone can lead to longer term depression of aggressive motivation, lasting at least 24 hours and that social interactions between crickets in grouped colonies may even have the potential to forge lifelong differences in individual behavioral “personalities”.

## Methods

### Experimental animals

Male Mediterranean field crickets, *Gryllus bimaculatus* (de Geer), were obtained from a commercial breeding stock (HW Terra, Aurachtal, Germany). All experiments were performed at ambient room temperature (22–25°C) and comply with animal welfare regulations in Germany. To minimize random differences in behavior due to daily variations in performance we always performed test and control experiments in parallel, avoiding times when aggression tends to be depressed (early afternoon and on generally dreary days, see [21, 22]). Our analysis is based on data gathered from interactions between 1576 pairs of male crickets. We defined basically 3 different groups depending on their experience prior to testing:

#### Short-term isolates (STI)

These crickets were obtained as young adults and first kept in our animal housing facility for 4–6 days as groups of 18–22 individuals under standard conditions (22–24°C, relative humidity 40–60%, 12–12 h light-dark regime daily feeding on bran and fresh vegetables, see [23]). Animals from this stock were then taken and kept isolated in individual glass jars with ample food for 48 h prior to experimentation.

#### Long-term isolates (LTI)

These crickets were obtained as last and last but one instar larvae and immediately isolated in individual glass jars, under the same conditions as STIs, for 6–8 days as larvae and for a further 10–14 days after the final moult as adults. They were thus kept in isolation for at least 16 to maximally 22 days.

#### Hyper-aggressive

These crickets served as standard opponents, against which the aggressiveness of STI and LTI crickets were evaluated. They were selected as noticeably larger individuals (> 5% heavier than test crickets) from the stock of STIs. Prior to testing, they were induced to fly for 3 min by suspending them in a warmed wind stream from a commercial grade air dryer, a treatment that markedly enhances aggression [24]. Due to this treatment and their larger size they win practically all fights against test crickets; in the rare cases when not the data were not evaluated.

### Evaluation of aggression and multiple defeat

**Fig 1:**
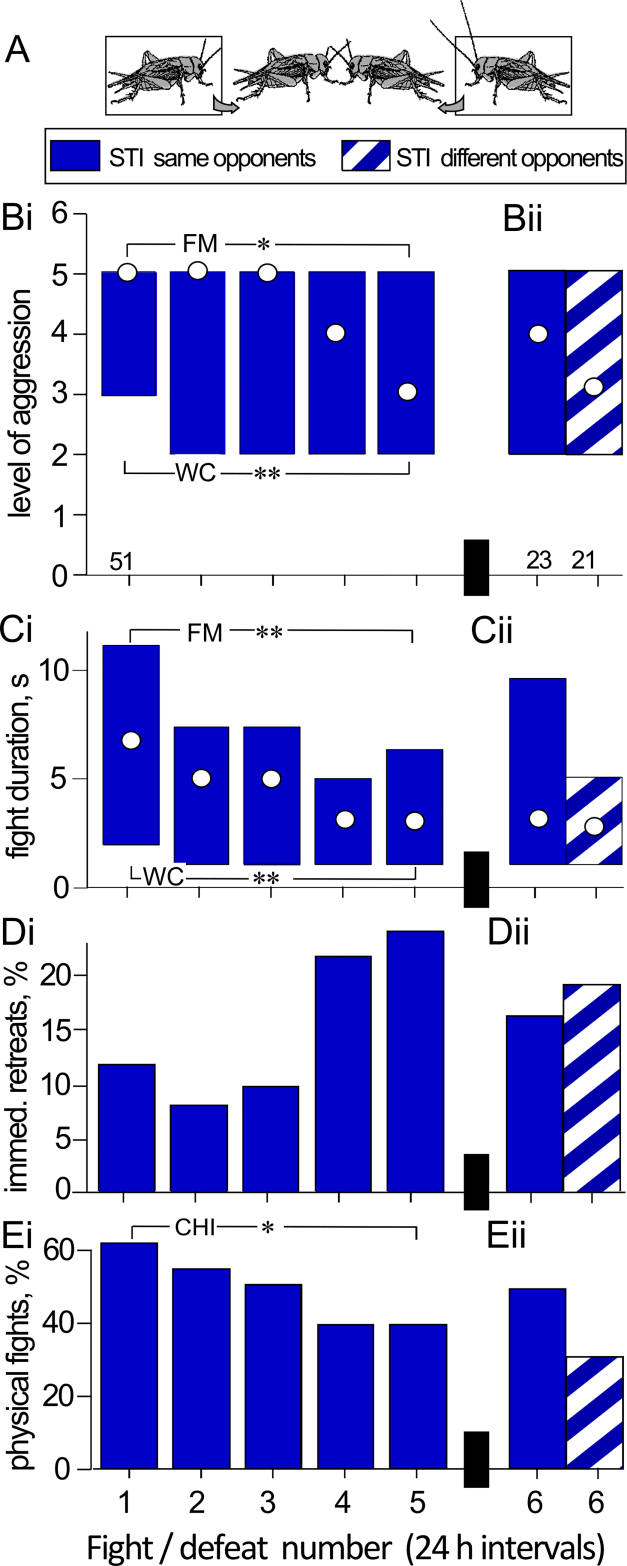
Aggressiveness of STI crickets that experienced 5 defeats at 24 h intervals against STI rivals. **(A)** The experimental paradigm. **(Bi)** Bar charts giving the level of aggression and **(Ci)** fight duration (circles M, bars IQR). **(Di)** Percentages of immediate retreats (level 1) and **(Ei)** physical fights (level 5 and 6). **(Bii-Eii)** As for Bi-Ei but for only half the previous losers that were re-matched against the same rivals (blue bars) and the remainder against different rivals (hatched blue bars). Significant differences between data sets as given by the Friedman test (FM) are indicated with asterisks, as are significant differences between the 1^st^ and 5^th^ fight as given by the Wilcoxon-signed rank test (WC) and chi-square test (CHI): *, ** P < 0.05, 0.01 respectively. N is giving above the x-axis in B.

**Fig 2:**
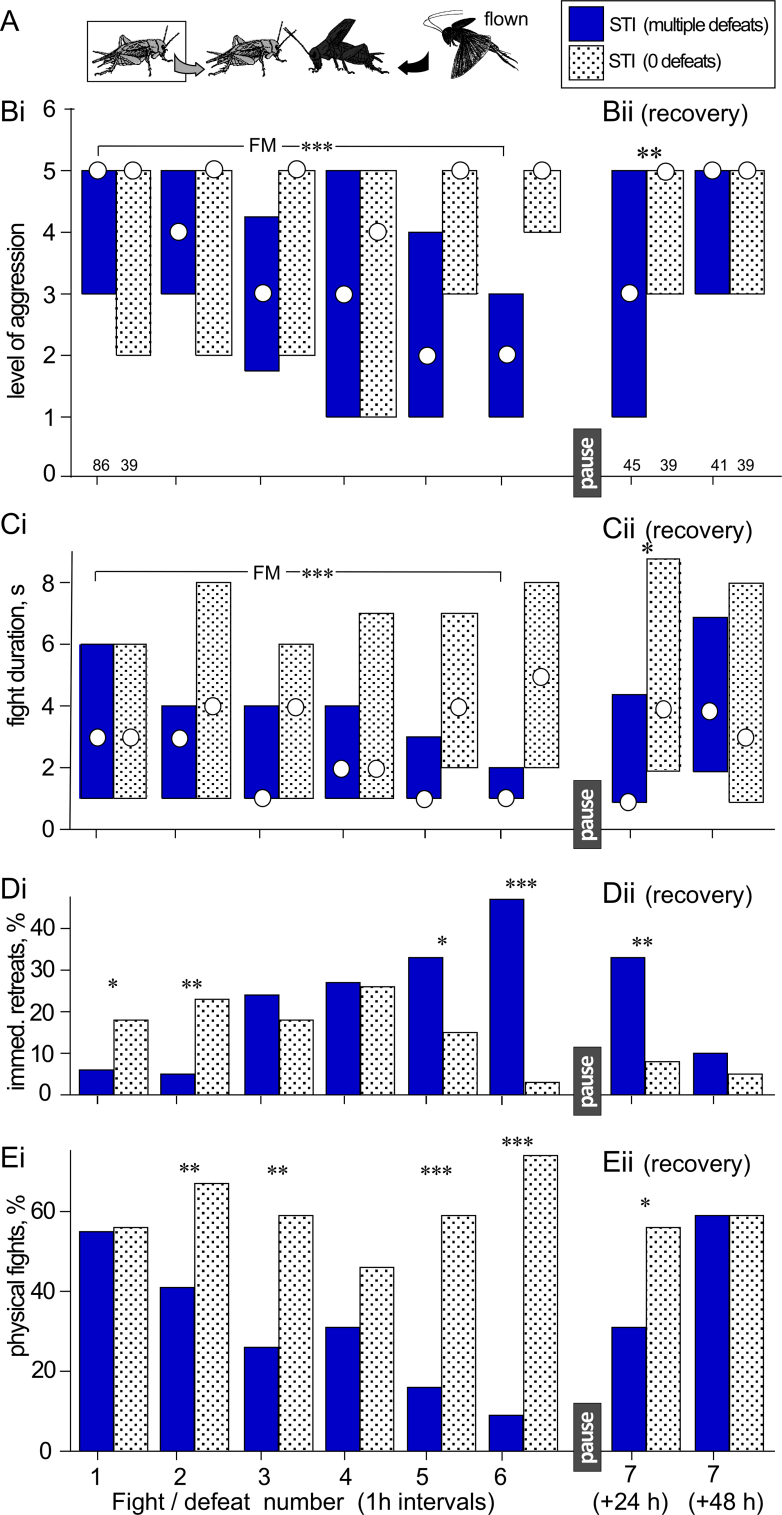
Aggressiveness of STI crickets that experienced 6 defeats at 1 h intervals against hyper-aggressive opponents. **(A)** Experimental paradigm with legend. **(Bi)** Bar charts giving the level of aggression and **(Ci)** fight duration (circles M, bars IQR) for STI crickets that suffered multiple defeats against hyper-aggressive opponents (blue bars) compared to the performances of fresh STI crickets at each match that suffered no previous defeat (stippled bars). **(Di)** As in B1, but giving percentages of immediate retreats (level 1) and **(Ei)** physical fights (level 5 and 6). **(Bii-Eii),** as for Bi-Ei but 24 h and 48 h after the previous defeat. Significant differences between data sets as given by the Friedman test (FM) are indicated with asterisks, as are significant differences between individual bars as given by the Wilcoxon-signed rank test: *, **, *** P < 0.05, 0.01, 0.001 respectively. N is giving above the x-axis in B. Note the maintained depression of aggression 24 h after experiencing multiple defeats in the test group.

**Fig 3:**
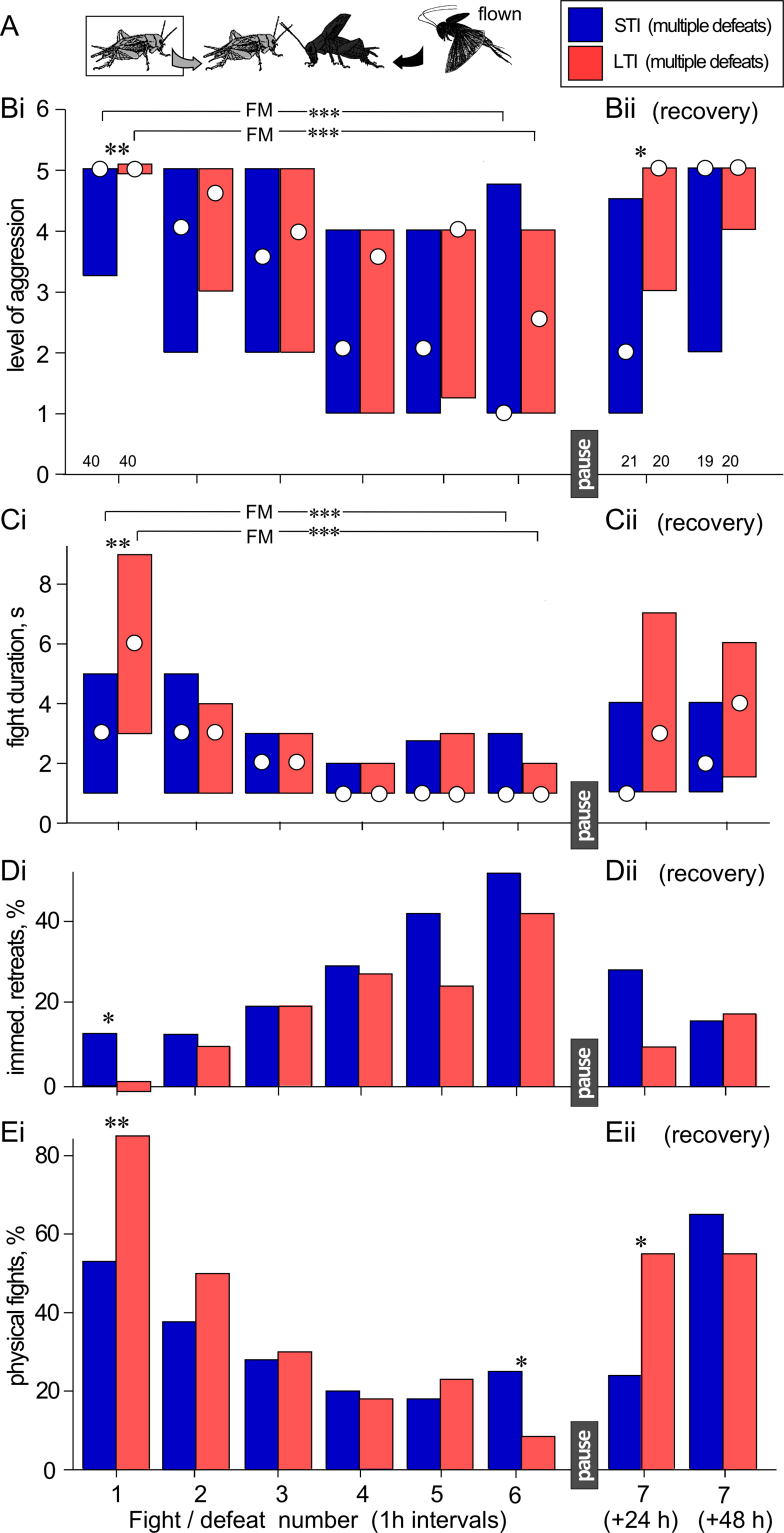
Comparison of the aggressiveness of STI and LTI crickets that experienced successive defeats at 1h intervals against hyper-aggressive opponents. **A)** Pictogram of the experimental paradigm with legend. **(Bi)** Level of aggression and **(Ci)** fight duration (circles M, bars IQR) for STI and LTI crickets (blue bars, red bars respectively) matched repeatedly against hyper-aggressive opponents. **(Di)** As in B1, but giving percentages of immediate retreats (level 1) and **(Di)** physical fights l (level 5 and 6). **(Aii-Dii)** As for Bi-Ei, but for contests staged 24 h and 48 h after the last defeat. Significant differences between data sets as given by the Friedman test (FM) are indicated with asterisks, as are significant differences between individual bars as given by the Wilcoxon-signed rank test: *, **, *** P < 0.05, 0.01, 0.001 respectively. N is giving above the x-axis in B. Note the higher aggression of LTI crickets at the first fight and their earlier recovery 24 h after suffering multiple defeat.

Aggressive behavior was evaluated in dyadic contests (see [25]) in which the test crickets were either weight-matched (< 5% weight difference; data Fig 1, Table 1) or 5% lighter when matched against standard hyper-aggressive opponents (data in Figs 2 and 3, Tables 2 and 3). In each case the two opponents were placed at opposite ends of a small, Perspex-glass rectangular fighting arena (l. w. h.: 16 x 9 x 7 cm) having a sand-covered floor and divided halfway along its length by an opaque sliding door. On removing the door, the crickets generally interact with each other within seconds. Their interactions follow an escalating sequence of stereotyped motor performances [22, 25], which do not differ significantly from fights that occur in the field as part of their normal behavioral repertoire [26]. The intensity of aggressiveness was scored on a scale of 0–6 [22, 24] denoting the level to which a fight escalates before the winner is established by the retreat of one contestant: Level 0: mutual avoidance without aggression. Level 1: one cricket attacks, the other retreats. Level 2: antennal fencing, whereby the contestants face each other and lash each other's antennae. Level 3: one contestant spreads its mandibles in a threat display. Level 4: mandible spreading by both crickets. Level 5: mandible engagement, whereby the two opponents interlock their mandibles. Level 6: grappling, an all-out fight involving repeated mandible engagements with biting, and body flipping. A contest can be concluded at any level by the retreat of one opponent. To check that a contest was indeed settled, we re-matched the same opponents < 3 min afterwards in a “loser-test”. In concluded fights, losers retreat immediately on contact, while the winner typically generates the characteristic rival song and body jerking movements [14]. Fights that were not concluded were not evaluated. We also evaluated the relative frequency of immediate retreat (level 1) and physical fighting (levels 5 and 6) exhibited by the losers. Fight duration, from first contact until establishment of the winner and loser, was measured to the nearest second with a stopwatch. Very occasionally, the animals appeared to lose contact with each other so that fighting paused for a brief period before resuming when contact was regained. As in previous studies, we chose to deduct the duration of these pauses in the few cases in which they occur in order to give a more representative measure of the actual time spent fighting.

**Table 1:** Data for STIs matched against each other 5 times in succession at 24 h intervals. The level and duration of aggression as well as percentages of immediate retreats and physical fights is given only for the 1^st^ and 5^th^ fights and also for a 6^th^ fight staged 24 h later against either the same or an unfamiliar opponent (same opp., diff. opp.): 50%, 25–75%, 10–90% give the median, IQR and 10 and 90 percentiles respectively.

	STI				statistics		
**level**	**50%**	**25–75%**	**10–90%**	**N**_**total**_	**P**	**W**	**U**
1^st^ fight	5	3–5	1–6	51			
5^th^ fight	3	2–5	1–5	51	0.001	398	
6^th^, same opp.	4	2–5	1–5	25			
6^th^, diff. opp.	5	2–5	1–5	26	0.315		273
**duration**	**50%**	**25–75%**	**10–90%**	**N**_**total**_	**P**	**W**	**U**
1^st^ fight	7	2–11	1–22	51			
5^th^ fight	3	1–7	1–11	51	0.001	521	
6^th^ fight same opp.	3	1–9	1–23	25			
6^th^ fight diff. opp.	2.5	1–5	1–15	26	0.535		292
**Immediate retreats**	**%**		**N**	**N**_**total**_	**P**	**X**^**2**^	
1^st^ fight	12		6	51			
5^th^ fight	24		12	51	0.114	2.487	
6^th^ fight same opp.	16		4	25			
6^th^ fight diff. opp.	19		5	26	0.079	0.778	
**physical fights**	**%**		**N**	**N**_**total**_	**P**	**X**^**2**^	
1^st^ fight	61		31	51			
5^th^ fight	39		20	51	0.026	4.936	
6^th^ fight same opp.	48		12	25			
6^th^ fight diff. opp.	31		8	26	0.214	1.543	

N_total_ gives the number of all interactions and N the number of immediate retreat and physical fights. Test statistics and P are given from Wilcoxon-, Mann-Whitney- and Chi-square tests (U, W, X^2^, P—boldface when significant).

**Table 2:** Data for STIs matched against standard hyper-aggressive opponents 5 times in succession at 1 h intervals compared to STIs at each trial that suffered no previous defeat. The level and duration of aggression as well as percentages of immediate retreats and physical fights is given for the 1^st^ and 6^th^ fights and also for a 7^th^ fight staged either 24 h or 48 h later (recovery 24 h, 48 h): 50%, 25–75%, 10–90% give the median, IQR and 10 and 90 percentiles respectively.

	STI				STI_control				statistics	
**level**	**50%**	**25–75%**	**10–90%**	**N**_**total**_	**50%**	**25–75%**	**10–90%**	**N**_**total**_	**P**	**U**
1st fight	5	3–5	2–5	86	5	2–5	1–5	39	0.533	1569
6th fight	2	1–3	1–4	86	5	4–5	2–5	39	**< 0.001**	**358**
recovery 24h	3	1–5	1–5	45	5	3–5	2–5	39	**0.002**	**547**
recovery 48h	5	3–5	1–5	41	5	3–5	2–5	39	0.919	789
**duration**	**50%**	**25–75%**	**10–90%**	**N**_**total**_	**50%**	**25–75%**	**10–90%**	**N**_**total**_	**P**	**U**
1st fight	3	1–6	1–11	86	3	1–6	1–16	39	0.704	1607
6th fight	1	1–2	1–4	86	5	2–8	1–12	39	**< 0.001**	**641**
recovery 24h	1	1–4	1–11	45	4	2–9	1–16	39	**0.021**	**629**
recovery 48h	4	2–7	1–16	41	3	1–8	1–16	39	0.589	743
**immediate retreats**	**%**		**N**	**N**_**total**_	**%**		**N**	**N**_**total**_	**P**	**X**^**2**^
1st fight	6		5	40	18		7	40	**0.036**	**4.393**
6th fight	47		40	40	3		1	40	**< 0.001**	**23.39**
recovery 24h	33		15	21	8		2	20	**0.005**	**7.765**
recovery 48h	10		4	19	5		1	20	0.397	0.714
**Physical fights**	**%**		**N**	**N**_**total**_	**%**		**N**	**N**_**total**_	**P**	**X**^**2**^
1st fight	55		47	40	56		22	40	0.917	0.010
6th fight	9		8	40	74		30	40	**<0.001**	**54.74**
recovery 24h	31		14	21	56		11	20	**0.020**	**5.339**
recovery 48h	59		24	19	59		12	20	1	**0**

N_total_ gives the number of all interactions and N the number of immediate retreat and physical fights. Test statistics and P are given from Mann-Whitney- and Chi-square tests (U, X^2^, P—boldface when significant).

**Table 3:** Data for STIs and LTIs matched 5 times in succession at 1 h intervals against standard hyper-aggressive opponents. The level and duration of aggression as well as percentages of immediate retreats and physical fights is given for the 1^st^ and 6^th^ fight and also for a 7^th^ fight staged either 24 h or 48 h later (recovery 24 h, 48 h): 50%, 25–75%, 10–90% give the median, IQR and 10 and 90 percentiles respectively.

	STI				LTI				statistics	
**level**	**50%**	**25–75%**	**10–90%**	**N**_**total**_	**50%**	**25–75%**	**10–90%**	**N**_**total**_	**P**	**U**
1st fight	5	3–5	1–5	40	5	5–5	4–5	40	**0.001**	**521**
6th fight	1	1–5	1–5	40	2	1–4	1–4	40	0.700	762
recovery 24h	2	2–4	1–5	21	5	3–5	1–5	20	**0.020**	**124**
recovery 48h	5	2–5	1–5	19	5	4–5	1–5	20	0.837	183
**duration**	**50%**	**25–75%**	**10–90%**	**N**_**total**_	**50%**	**25–75%**	**10–90%**	**N**_**total**_	**P**	**U**
1st fight	3	1–5	1–9	40	6	3–9	2–14	40	**0.001**	**467**
6th fight	1	1–3	1–5	40	1	1–2	1–3	40	0.761	773
recovery 24h	1	1–4	1–6	21	3	1–7	1–26	20	0.078	144
recovery 48h	2	1–4	1–5	19	4	1–6	1–13	20	0.058	123
**Immediate retreats**	**%**		**N**	**N**_**total**_	**%**		**N**	**N**_**total**_	**P**	**X**^**2**^
1st fight	13		5	40	0		0	40	**0.018**	**5.561**
6th fight	53		21	40	43		17	40	0.370	0.801
recovery 24h	29		6	21	10		2	20	0.126	2.334
recovery 48h	16		3	19	15		3	20	0.931	0.007
**Physical fights**	**%**		**N**	**N**_**total**_	**%**		**N**	**N**_**total**_	**P**	**X**^**2**^
1st fight	53		21	40	85		34	40	**0.001**	**9.574**
6th fight	25		10	40	8		3	40	**0.040**	**4.195**
recovery 24h	24		5	21	55		11	20	**0.042**	**4.133**
recovery 48h	58		11	19	65		13	20	0.653	0.201

N_total_ gives the number of all interactions and N the number of immediate retreat and physical fights. Test statistics and P are given from Mann-Whitney and Chi-square tests (U, X^2^, P—boldface when significant).

The effects of multiple defeats were investigated by re-matching test crickets repeatedly at either 1 h or 24 h intervals, ensuring that each contest was settled in a loser-test. In cases where the inter-fight interval was 24 h, test crickets were gently transferred back to their glass jars after each fight and supplied with ample moist food until the next trial. Previous studies have eliminated the possibility that gentle handling influences subsequent aggressive behavior of crickets [27].

### Statistical analysis

All statistical tests were performed using standard commercial software (Prism 5, GraphPad Software Inc, La Jolla, CA, USA) running on a personal computer (Dell T3500, USA). The median (M) and the interquartile range (IQR) were calculated for all data sets. To test for significant differences in aggression with successive fights we applied the Friedman test for paired data sets and the Kruskal-Wallis test for unpaired data sets. Differences between specific groups with non-normally distributed data sets were tested using the Wilcoxon-signed rank test for paired data sets and the Mann-Whitney U test for unpaired data. The Chi-Square test was employed to test for differences in the frequency of immediate retreats and physical fights. Student’s unpaired t-test was used to test for differences in weight between multiple winners and losers as these data were normally distributed. The significance level was set at < 0.05 for comparing two groups.

## Results

### Multiple defeat at 24 h intervals in short-term isolates (STI)

In our first experiment with STIs, we found that losers of an initial fight tended to lose again against the same previous opponents when matched 24 h later. Thus, from a group of 102 individual STI crickets that suffered their first defeat, the majority lost again at the next encounter against the same opponent 24 h later (76%, N = 78, data not illustrated). This trend continued so that at the 5^th^ fight and after 4 previous defeats, 50% (N = 51) of the original cohort of 102 losers had lost 5 fights in succession against the same opponent. Since these persistent losers were not significantly different in weight to the persistent winners, fight outcome was not due to weight asymmetry (mean, standard deviation winners = 0.721, 0.099 g, losers: 0.731, 0.097 g; unpaired t-test: N = 51, T = 0.511, P = 0.609, not significant).

We next analyzed the aggressiveness of these 51 persistent losers at all their previous fights (Fig 1, Table 1 for statistical details). Corroborating earlier studies [22, 23, 25, 28, 29], the initial fights (0 previous defeats) of weight-matched male crickets after an isolation period of 24 h typically escalate to level 5 (median M, interquartile rang IQR = 5, 3–5; Fig 1Bi) and last several seconds (M, IQR = 7, 2–11 s; Fig 1Ci). Each of these fights generated clear losers which subsequently retreated from their previous opponents at the “loser-test” staged less than 3 min after defeat. As shown in earlier studies, the losers of a single previous fight engage again in physical fighting against the same opponents when tested at a 2^nd^ fight 24 h later (level: M, IQR = 5, 2–5, duration: M, IQR = 5, 1–8 s). However, with each further defeat there was a clear reduction in aggression in terms of escalation level and fight duration (Friedman tests, FM: P level = 0.016, P duration < 0.001; Fig 1Bi and 1Ci). Thus, at the 5^th^ fight the level of aggression and fight duration were both significantly lower than at the first fight (level: M, IQR = 3, 2–5, duration: M, IQR = 3, 1–7 s; level: W = 398, N = 51, P < 0.001; duration: W = 521, N = 51, P < 0.001). Furthermore, the relative frequency of immediate retreats exhibited by the losers on confronting the opponents increased from 12% at the 1^st^ fight to 24% at the 5^th^, but this was not statistically significant (Chi-square test: X^2^ = 2.487, N = 51, P = 0.114; Fig 1Di, Table 1), whereas the frequency of actual physical interactions decreased significantly from 61% at the 1^st^ fight, to 39% at the 5^th^ fight (X^2^ = 4.936, N = 51, P = 0.026; Fig 1Ei).

To test whether the reduction in aggression of persistent losers is an adaptive strategy towards a familiar opponent, we staged an additional (6^th^) fight 24 h later, at which approximately half the losers of the 5^th^ fight were re-matched against the same winners as in all previous fights, while the remainder were matched against a different winner. As illustrated in Fig 1Bii–1Eii, the persistent losers matched against the same winners were not less aggressive than those matched against different winners (Mann-Whitney U tests, level: U = 273, N1 = 25, N2 = 26, P = 0.315; duration: U = 292.5, P = 0.535; immediate retreats: X^2^ = 0.778, P = 0.079, physical fights: X^2^ = 1.543, P = 0.214). If anything, multiple losers matched against the familiar rival tended to be more aggressive, in that they escalated more often to physical fights, but this was not statistically significant.

### Multiple defeat at 1 h intervals in short-term isolates (STI)

We next evaluated the effect of multiple defeats at shorter intervals (1 h). To completely eliminate the possibility of differences in aggression arising due to recognition of a familiar opponent, the test crickets were matched at each trial against a different “standard hyper-aggressive” opponent (flown for 3 min. prior to test; see [24, 30]). Each of these interactions were compared to those of fresh STIs, which experienced no previous defeats, matched at the same time against standard hyper-aggressive opponents (Fig 2, Table 2).

The test crickets, which suffered defeat at each successive fight showed a progressive reduction in aggression (same animals at each trial: FM, level: P < 0.001, duration: P < 0.001), whereas the control crickets, which were different fresh isolates at each trial, always exhibited physical fighting (level 5) with no difference in aggression at each trial (Kruskal-Wallis tests, KW, level: P = 0.132, duration: P = 0.522, Fig 2Bi and 2Ci, Table 2). Thus, test crickets at the 5^th^ fight, that had experienced 4 previous defeats, rarely fought the hyper-aggressive opponent (level: M, IQR = 2, 1–3; duration: M, IQR = 1, 1–2 s; significantly different to controls: level: U = 358, N1 = 39, N2 = 86, P < 0.001; duration: U = 641, N1 = 39, N2 = 86, P < 0.001). This trend is also illustrated in Fig 2Di, which shows that the test crickets became increasingly more likely to retreat immediately on confronting the hyper-aggressive opponent with each successive defeat, and in Fig 2Ei which shows that the test crickets become progressively less likely to engage in physical interactions (immediate retreats: X^2^ = 23.397, P < 0.001; physical fights: X^2^ = 54.747, P < 0.001; Table 2).

Since crickets require at least 3 h to recover from a previous defeat [17, 23], it is not so surprising that they show depressed aggression after multiple defeats at 1h intervals. More relevant to this experiment, however, is the finding that multiple losers still showed depressed aggressiveness when tested 24 h after the last defeat (Fig 2Bii–2Eii). Compared to controls that suffered one defeat and fully recovered their aggressiveness after 24 h (level: M, IQR = 5, 3–5, duration: M, IQR = 4, 2–9 s), test crickets that suffered 6 previous defeats escalated less at the 7^th^ fight (level: M, IQR = 3, 1–5; difference to control: U = 547.5, N1 = 45, N2 = 39, P = 0.002) and interacted significantly shorter periods of time (M, IQR = 4, 2–9 s: difference to controls: U = 629, N1 = 45, N2 = 39, P = 0.021; Fig 2Bii–2Eii). Furthermore, after the 24 h recovery time, the multiple losers exhibited immediate retreat more often than controls (test 33%, control 8%, X^2^ = 7.765, P = 0.005, Fig 2Dii), and less often physical fighting (test 31%, control crickets 56%, X^2^ = 5.339, P = 0.020; Fig 2Eii; Table 2). In contrast to this, multiple-defeated crickets given 48 h to recover from their last defeat appeared to have recovered their aggressiveness. For example, after 48 h recovery, neither the level nor duration of aggression was significantly different to control crickets (level: U = 789.5, N1 = 41, N2 = 39, P = 0.919, duration: U = 743.5, N1 = 41, N2 = 39, P = 0.589) and there were also no significant differences in the frequency of immediate retreat or physical interactions (immediate retreats: X^2^ = 0.714, P = 0.397; physical interactions: X^2^ = 0, P = 1).

### Multiple defeat in long-term isolates (LTI)

Since a series of defeats at 1 h intervals can lead to longer term depression of aggression lasting at least 24 h (Fig 2), we next tested whether STIs may be influenced by earlier social experiences. To do this, we compared the aggressive behavior STIs to that of LTIs. As in the previous experiment, these crickets were also matched against a different, standard hyper-aggressive opponent at each contest (Fig 3, Table 3).

Interestingly, the LTIs were significantly more aggressive than the STIs at their very first fight (LTIs, level: M, IQR = 5, 5–5; duration: M, IQR = 6, 3–9 s; STIs level: M, IQR = 5, 3.25–5, duration: M, IQR = 3, 1–5 s; U level = 521, N1 = N2 = 40, P < 0.001, U duration = 467, P < 0.001; Fig 3Bi and 3Ci). This is also illustrated by the fact that none of the LTIs retreated on first confronting their hyper-aggressive opponents, compared to 13% immediate retreats for STIs (X^2^ = 5.561, P = 0.018, Fig 3Di). Furthermore, 85% of the LTIs engaged in physical fighting compared to 53% for STIs (X^2^ = 9.574, P = 0,001, Fig 3Ei).

After this first fight, the aggressiveness of both LTIs and STIs declined with each successive defeat (trials 2–6) and there were no longer any differences in the aggressive behavior between the two groups (FM: STI level: P < 0.001, duration: P < 0.001; LTI level: P < 0.001, duration: P < 0.001). Despite this, the LTIs appeared to be less susceptible to the effect of multiple defeat than STIs. Thus, 24 h after the last defeat, LTIs appeared to have partly regained their aggressiveness and were significantly more aggressive than the STIs with respect to their level of aggression (LTIs M, IQR = 5, 3–5; STIs M, IQR = 2, 1–4.5, U = 124, N1 = 21, N2 = 20, P = 0.020; Fig 3Bii) and frequency of aggressive interactions (immediate retreats: X^2^ = 2.334, P = 0.126, Fig 3Dii; physical interactions: X^2^ = 4.133, P = 0.042, Fig 3Eii). However, 48 h after the last defeat STIs and LTIs showed high levels of aggression, and there was no longer any difference between them (LTI level: M, IQR = 5, 4–5, duration: M, IQR = 4, 1.5–6 s; STI level: M, IQR = 5, 2–5, duration: M, IQR = 2, 1–4 s; U level = 183, N1 = 20, N2 = 19, P = 0.837, U duration = 123.5, N1 = 20, N2 = 19, P = 0.058; Fig 3Bii–3Eii, Table 3).

## Discussion

This study revealed that chronic social defeat has a long-term influence on the behavior of adult male crickets (*Gryllus bimaculatus*). As in many other animals [1, 2], crickets that have lost a fight behave submissively towards any adult conspecific male, and require at least 3 h to regain their original aggressive status [16, 17, 23, 24]. Whereas losers of a first fight are equally aggressive towards the previous winners after 24 h, we observed that the losers tended to lose again at this second fight and also at subsequent contests (for similar findings see [21] on *Gryllus integer*, [31] on *Acheta domesticus* and [20] on *Drosophila*). In addition, we found that losers that lost repeatedly against the same opponents on 5 consecutive days became progressively less aggressive with each defeat (Fig 1). There are several feasible explanations for these observations which must be considered before any conclusions can be drawn.

Relative body mass is known to be a major determinant of fighting success in many animals including crickets [21, 31, 32]. However, this does not explain why losers lost repeatedly, since they were not found to weigh less than their opponents. We also considered the possibility that the losers may have adjusted their fighting strategy towards familiar opponents, as suggested for *Drosophila* [20]. When contestants recognise each other, aggression generally declines in subsequent interactions because relative dominance ranks have already been established [33, 34]. However, in species lacking individual recognition, aggression is not predicted to decline over subsequent encounters because the relative ranks of social partners are not clear unless individuals engage in new aggressive contests [35]. Nonetheless, notwithstanding the fact that some insects have the capacity to discriminate individual conspecifics [36], this does not seem to apply to crickets. One-time losers are known to retreat even from unfamiliar opponents [15, 23, 26, 27, 37] and here we found that persistent losers re-matched against different rivals were equally submissive as those matched against familiar opponents (Fig 1).

Another explanation could be that reduced aggression in losers is due to some aspect of the winner’s behavior. In crayfish, for example, pheromone signals in the urine released by a dominant male acts on opponents to reduce the duration of future aggressive interactions [38]. Male dominance also influences pheromone expression in the Australian field cricket *Teleogryllus oceanicus* [39]. However, as far as presently known, cuticular pheromones in male crickets (see Tregenza, [40]) act rather to initiate [41, 42] and promote [43] aggression, rather than suppress it. Nonetheless, since winner crickets become more aggressive [14] and losers assess their opponent’s actions for the decision to flee [27], a winner’s augmented agonistic efforts could induce their opponents to give up earlier. However, winner-effects, which are widespread in the Animal Kingdom [16, 2], are presently not known to last longer than 20 min in crickets [14] and are thus unlikely to have influenced the losers when re-matched at 24 h intervals as in the present study.

Despite the above reasoning, and to strengthen our arguments, we sought to eliminate any potential influences of the winner-effect and individual recognition by evaluating the performances of test crickets that suffered multiple defeats at 1 h intervals against different hyper-aggressive opponents at each successive contest (Fig 2). Since the losers in this experiment were allowed less than the 3 h required by crickets to recover from social defeat [17, 23], it is perhaps not so surprising that their fighting efforts declined with each match. Of more significance was the finding that after 6 multiple defeats at 1 h intervals the losers exhibited a long-term depression of aggression that lasted longer than 24 h (Fig 2). Considering all arguments, the most parsimonious explanation for this is that losing has some residual effect, lasting at least 24 h, that will accumulate when repeated defeats are experienced within a given time frame, and that this leads to an extension of the loser effect, i.e. prolonged depression of aggressive motivation in subordinates. Previous workers have noted that three consecutive defeats can extend the duration of the loser effect in crickets for up to 6 h [16], but longer term loser-depression resulting from defeat and lasting over a whole day has never been observed.

In our present paradigms, the crickets appear to have recovered from chronic social defeat after 48 h. Despite this, our findings with long term isolated (LTI) crickets, which were kept individually from that last larval stages onwards until testing at sexual maturity, suggest that social interactions may have even longer, possibly lifelong, effects on subsequent behavior. For example, compared to short-term isolates (STIs, 48 h), LTIs (16–22 days) were significantly more aggressive at their very first fight as adults (Fig 3). None retreated on sighting the hyper-aggressive opponent, and the large majority (85%) engaged in physical fighting (levels 5 and 6). This is most unusual. To our knowledge, such homogeneity has never been observed in the aggressiveness of crickets, of which a significant proportion (10–20%) typically retreat immediately on contacting a conspecific male (see also Fig 1Di). Hence, crickets without any fighting experience as adults are highly aggressive and less variable in their fighting behavior. Furthermore, the LTIs were far less susceptible to multiple defeats, in that they were more aggressive than STIs 24 hours after 6 successive defeats. Numerous studies have noted that individuals reared in isolation have higher aggression [1, 15, 44] and isolation is often viewed as a pathological condition that can lead to increased aggressiveness [45]. Experiments in crickets, however, have demonstrated that isolation allows the animals to escape from social subjugation by a few dominant individuals in a group and recover to a state of heightened aggressiveness, which in crickets is considered to be the natural default condition [23]. Accordingly, LTIs are more aggressive and less prone to the effects of social defeat because they have had practically no previous adverse social agonistic experience as adults. It should also be noted, that larval crickets also engage in low level agonistic interactions [46], but it is not known whether they form stable dominant-subordinate relationships with possible consequence for the future behavior of adults.

Social experiences gained under grouped conditions typical for cricket breeding colonies will probably not only affect aggressive behavior. The experiences of losing 2 consecutive fights in short-term isolates (48 h) is known to result in reduced general motility and exploratory behavior, whereas winning has the opposite effects [13]. In this earlier study, similar behavioral differences were also evident between the future winners and future losers 24 h before the fights were staged, indicating that earlier social experience could shape inter-individual behavioral differences in crickets (see also [47] on *Gryllus integer*). Our current and earlier study thus support the notion of “personality” in invertebrates being both a cause and consequence of contest behavior [10].

In mammals, including humans, social defeat, is generally regarded as a major stressor and risk factor for neuropsychiatric disorders such as depression [6–8]. In rodents, for example, repeated intermittent social defeat leads to reduced locomotor activity and elevated blood levels of stress hormones (cortisol and norepinephrine), but lower brain levels of the amines dopamine and serotonin [5]. In insects, the biogenic amine octopamine, the invertebrate analogue of noradrenaline [48], is considered to be a major stress hormone (reviews: [49, 50]), that is released in crickets during fighting behavior [51] and in response to repeated fight or flight stress, such as repeated exposure to a mock predator [52]. Similar to the effects of social defeat on cricket motility [13], exposure to a mock predator also leads to reduced locomotor activity and increased shelter-seeking behavior, and these effects could be mimicked by octopamine treatment [53]. However, as far as is known in crickets, octopamine appears to have a promoting, rather suppressing effect on aggression [14, 25, 43]. Clearly, future investigations must therefore address the role of neuromodulators for establishing long-term behavioral depression following chronic social defeat.
